# Supplementary material for: Machine Learning Accelerates Crystallization for Structure Determination
Source: Angew Chem Int Ed Engl. 2026 May 4;65(25):e1218503. doi: 10.1002/anie.1218503 (PMC13266958; doi:10.1002/anie.1218503)

checkCIF/PLATON report

Structure factors have been supplied for datablock(s) lcz074

THIS REPORT IS FOR GUIDANCE ONLY. IF USED AS PART OF A REVIEW PROCEDURE FOR PUBLICATION, IT SHOULD NOT REPLACE THE EXPERTISE OF AN EXPERIENCED CRYSTALLOGRAPHIC REFEREE.

No syntax errors found. CIF dictionary Interpreting this report

Datablock: lcz074

Bond precision:	C-C = 0.0080 A		Wavelength=1.54184
Cell:	a=8.9202(4)	b=18.1007(8)	c=20.1319(9)
	alpha=90	beta=92.225(4)	gamma=90
Temperature:	100 K		

	Calculated	Reported
Volume	3248.1(3)	3248.1(3)
Space group	P 21/c	P 1 21/c 1
Hall group	-P 2ybc	-P 2ybc
Moiety formula	C15 H3 Ag3 F18 N6, C9 H9 Br O	C15 H3 Ag3 F18 N6, C9 H9 Br O
Sum formula	C24 H12 Ag3 Br F18 N6 O	C24 H12 Ag3 Br F18 N6 O
Mr	1145.91	1145.92
Dx,g cm-3	2.343	2.343
Z	4	4
Mu (mm-1)	17.102	17.102
F000	2176.0	2176.0
F000’	2183.35	
h,k,lmax	11,22,24	10,22,24
Nref	6475	6270
Tmin,Tmax	0.029,0.077	0.517,1.000
Tmin’	0.005	

Correction method= # Reported T Limits: Tmin=0.517 Tmax=1.000
AbsCorr = MULTI-SCAN

```
Data completeness= 0.968          Theta(max)= 72.873

R(reflections)= 0.0371( 5282)     wR2(reflections)= 0.1020( 6270)

S = 1.069                Npar= 480
```

The following ALERTS were generated. Each ALERT has the format
test-name_ALERT_alert-type_alert-level.
Click on the hyperlinks for more details of the test.

Alert level C

```
PLAT213_ALERT_2_C Atom F011           has ADP max/min Ratio .....        3.3 prolat
PLAT213_ALERT_2_C Atom F017           has ADP max/min Ratio .....        3.6 prolat
PLAT213_ALERT_2_C Atom F01F           has ADP max/min Ratio .....        3.5 prolat
PLAT234_ALERT_4_C Large Hirshfeld Difference F00Q     --C01H     .      0.17 Ang.
PLAT342_ALERT_3_C Low Bond Precision on  C-C Bonds ..............    0.00805 Ang.
PLAT911_ALERT_3_C Missing FCF Refl Between Thmin & STh/L=    0.600         6 Report
               -4 20  2,  -5 19  3,   3 13 13,   1 16 16,   3 10 20,  -1  0 24,
```

Alert level G

```
PLAT003_ALERT_2_G Number of Uiso or U(i,j)  Restrained non-H-Atoms         3 Report
PLAT083_ALERT_2_G SHELXL Second Parameter in WGHT  Unusually Large      7.00 Why ?
PLAT177_ALERT_4_G The CIF-Embedded .res File Contains DELU Records         1 Report
PLAT186_ALERT_4_G The CIF-Embedded .res File Contains ISOR Records         1 Report
PLAT192_ALERT_3_G A Non-default DELU Restraint Value for First Par    0.0010 Report
PLAT192_ALERT_3_G A Non-default DELU Restraint Value for SecondPar    0.0020 Report
PLAT242_ALERT_2_G Low    'MainMol' Ueq as Compared to Neighbors of      C014 Check
PLAT242_ALERT_2_G Low    'MainMol' Ueq as Compared to Neighbors of      C018 Check
PLAT242_ALERT_2_G Low    'MainMol' Ueq as Compared to Neighbors of      C01C Check
PLAT242_ALERT_2_G Low    'MainMol' Ueq as Compared to Neighbors of      C01D Check
PLAT242_ALERT_2_G Low    'MainMol' Ueq as Compared to Neighbors of      C01G Check
PLAT242_ALERT_2_G Low    'MainMol' Ueq as Compared to Neighbors of      C01H Check
PLAT434_ALERT_2_G Short Inter HL..HL Contact F00B     ..F01F     .      2.80 Ang.
                                    2-x,-1/2+y,3/2-z  =     2_746 Check
PLAT720_ALERT_4_G Number of Unusual/Non-Standard Labels ..........        65 Note
              Ag01    Ag02    Ag03    Br04    O005    F006    F007    F008
              F009    F00A    F00B    F00C    F00D    N00E    F00F    F00G
              N00H    F00I    N00J    N00K    N00L    N00M    F00N    F00O
              F00P    F00Q    C00R    H00R    C00S    H00S    C00T    C00U
              C00V    H00V    C00W    C00X    H00X    C00Y    C00Z    H00Z
              C010    F011    C012    H012    C013    C014    C015    C016
              F017    C018    C019    C01A    H01A    H01B    H01C    C01B
              C01C    C01D    C01E    H01D    H01E    H01F    F01F    C01G
              C01H
PLAT790_ALERT_4_G Centre of Gravity not Within Unit-Cell: Resd.  #         2 Note
              C9 H9 Br O
PLAT860_ALERT_3_G Number of Least-Squares Restraints ............         13 Note
PLAT883_ALERT_1_G Absent Datum for _atom_sites_solution_primary ..     Please Do !
PLAT910_ALERT_3_G Missing FCF Reflection(s) Below Theta(Min)[Deg]=      4.40 Note
                0  1  1,
PLAT912_ALERT_4_G Missing # of FCF Reflections Above STh/L=  0.600       188 Note
PLAT941_ALERT_3_G Average HKL Measurement Multiplicity ..........        4.0 Low
```

```
PLAT969_ALERT_5_G The 'Henn et al.' R-Factor-gap value ..........      3.044 Note
              Predicted wR2: Based on SigI**2  3.35 or SHELX Weight  9.54
PLAT978_ALERT_2_G Number C-C Bonds with Positive Residual Density.         1 Info
```

```
   0 ALERT level A = Most likely a serious problem - resolve or explain
   0 ALERT level B = A potentially serious problem, consider carefully
   6 ALERT level C = Check. Ensure it is not caused by an omission or oversight
  22 ALERT level G = General information/check it is not something unexpected

   1 ALERT type 1 CIF construction/syntax error, inconsistent or missing data
  13 ALERT type 2 Indicator that the structure model may be wrong or deficient
   7 ALERT type 3 Indicator that the structure quality may be low
   6 ALERT type 4 Improvement, methodology, query or suggestion
   1 ALERT type 5 Informative message, check
```

Validation response form

Please find below a validation response form (VRF) that can be filled in and pasted into your CIF.

```
# start Validation Reply Form
_vrf_PLAT213_lcz074
;
PROBLEM: Atom F011            has ADP max/min Ratio .....        3.3 prolat
RESPONSE: ...
;
_vrf_PLAT234_lcz074
;
PROBLEM: Large Hirshfeld Difference F00Q     --C01H     .       0.17 Ang.
RESPONSE: ...
;
_vrf_PLAT342_lcz074
;
PROBLEM: Low Bond Precision on  C-C Bonds ..............    0.00805 Ang.
RESPONSE: ...
;
_vrf_PLAT911_lcz074
;
PROBLEM: Missing FCF Refl Between Thmin & STh/L=    0.600          6 Report
RESPONSE: ...
;
# end Validation Reply Form
```

It is advisable to attempt to resolve as many as possible of the alerts in all categories. Often the minor alerts point to easily fixed oversights, errors and omissions in your CIF or refinement strategy, so attention to these fine details can be worthwhile. It is up to the individual to critically assess their own results and, if necessary, seek expert advice.

PLATON version of 17/09/2025; check.def file version of 17/09/2025

---

duplicate check

No duplication found

Datablock lcz074 - ellipsoid plot

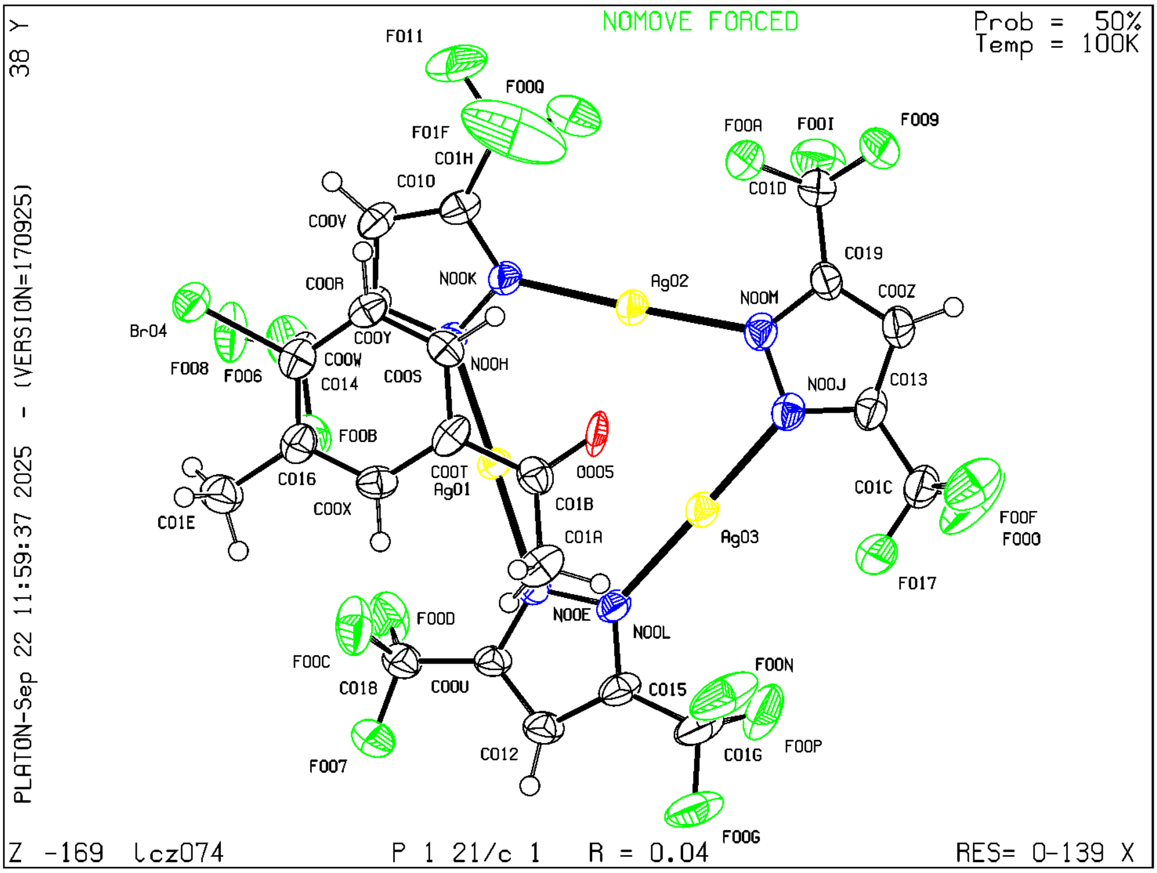

Supplement: Supplementary file 2 — Supporting File 2: anie72462‐sup‐0002‐DataFile.zip. [file ANIE-65-e1218503-s002.zip › supplementary_materials_crystallographic_data submit.zip!/cif_and_checkcif(1-114)/Ag3Pz3_74/Ag3Pz3_74_checkcif.pdf]
